# Crime Scene Reconstruction Using a Fully Geomatic Approach

**DOI:** 10.3390/s8106280

**Published:** 2008-10-08

**Authors:** Eros Agosto, Andrea Ajmar, Piero Boccardo, Fabio Giulio Tonolo, Andrea Lingua

**Affiliations:** 1 Politecnico di Torino, DITAG, C.so Duca degli Abruzzi, 24 – 10129 Torino, Italy; E-Mails: eros.agosto@polito.it; piero.boccardo@polito.it; andrea.lingua@polito.it; 2 ITHACA (Information Technology for Humanitarian Assistance, Cooperation and Action), Via P.C. Boggio, 61 - 10138 Torino, Italy; E-Mail: fabio.giuliotonolo@ithaca.polito.it

**Keywords:** Crime scene, GIS, footprint, human walk, bloodstain

## Abstract

This paper is focused on two main topics: crime scene reconstruction, based on a geomatic approach, and crime scene analysis, through GIS based procedures. According to the experience of the authors in performing forensic analysis for real cases, the aforesaid topics will be examined with the specific goal of verifying the relationship of human walk paths at a crime scene with blood patterns on the floor. In order to perform such analyses, the availability of pictures taken by first aiders is mandatory, since they provide information about the crime scene before items are moved or interfered with. Generally, those pictures are affected by large geometric distortions, thus - after a brief description of the geomatic techniques suitable for the acquisition of reference data (total station surveying, photogrammetry and laser scanning) - it will be shown the developed methodology, based on photogrammetric algorithms, aimed at calibrating, georeferencing and mosaicking the available images acquired on the scene. The crime scene analysis is based on a collection of GIS functionalities for simulating human walk movements and creating a statistically significant sample. The developed GIS software component will be described in detail, showing how the analysis of this statistical sample of simulated human walks allows to rigorously define the probability of performing a certain walk path without touching the bloodstains on the floor.

## 1. Introduction

The accurate recording of crime scene details is crucial for several reasons: first of all, it will provide investigators with information which they may not otherwise have knowledge of and, furthermore, it will assist the court in reconstructing the scene [6]. Nowadays, high geometric accuracy 3D crime scene reconstructions based on geomatic techniques are frequently used for forensic investigations [2, 4-5], since evidence gathered with topographic and photogrammetric devices can be more compelling for juries and allow investigators to virtually “revisit” a crime scene.

This paper deals with crime scene mapping techniques based on a geomatic approach and the subsequent crime scene analysis through GIS based procedures. The described crime scene reconstruction refers to a real experience, but since the investigation phase is still ongoing, clear references to the fact cannot be provided (therefore all the pictures, figures and results will be made anonymous for legal issues).

According to this preliminary remark and in order to clearly identify the goals of this specific crime scene analyses, it has to be stated that:

- the specific aim of the procedures described in this paper is to verify the compatibility of the walk paths performed on the crime scene by a suspect, with regards to the bloodstains present on the ground;
- the object of the crime scene mapping, for this specific work, is a floor covered by bloodstains.

The methodology, based both on geomatic survey techniques and GIS analyses tools developed to achieve the aforesaid goals, requires:

- the availability of pictures taken by first aiders (law enforcement agents, forensic scientists, etc.), that provides information about the crime scene before items are moved or interfered with. Generally those pictures are acquired for record keeping purposes, focusing the attention on the semantic content and neglecting the geometric one, thus they are characterized by large geometric distortions that require an adequate modelling to be corrected;
- the availability of the shoes of the suspect (eventually of all the people whose movements on the crime scene have to be verified), to create a sole 3D model required as input data to reconstruct a specific sole footprint. The availability of the real shoes is critical because particular sole consumption shapes may affect significantly shape and extent of the contact surface.

**Figure 1.**
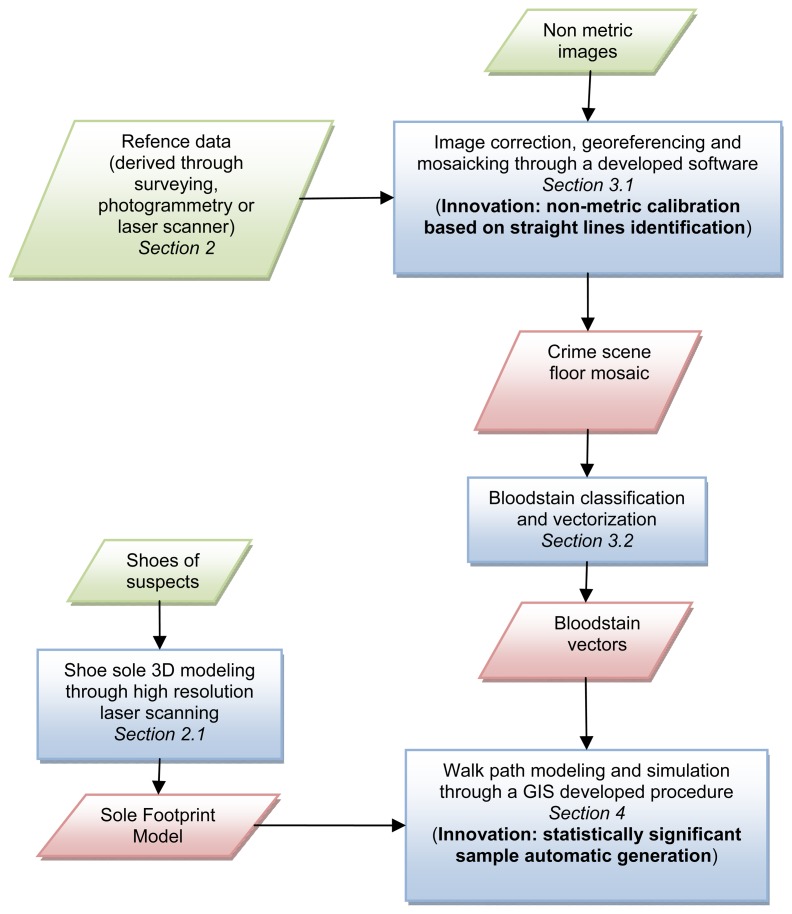
Schema of the developed methodology.

A schema of the methodology developed by the authors is proposed in Figure 1, to allow the readers to better understand the structure of this paper and its most innovative parts.

## 2. Reference data acquisition

In order to correctly georeference all the images and data acquired at a crime scene by first aiders it is fundamental to have the sufficient number of 3D reference points characterized by high geometric accuracy. These point can be used both as Ground Control Points (GCPs), required for the georeferencing procedures (see Section 3.1.2), and as independent Check Points (CPs), useful for the evaluation of accuracy. Generally this kind of data are obtained by means of surveying or phogrammetry. In the following chapters these techniques will be briefly described; they are often used jointly in order to have a redundant number of measurements, for two main reasons: crime scenes are not easily accessible in case of accidental data loss and the availability of redundant data allows one to evaluate the accuracy of the estimated coordinates.

**Figure 2.**
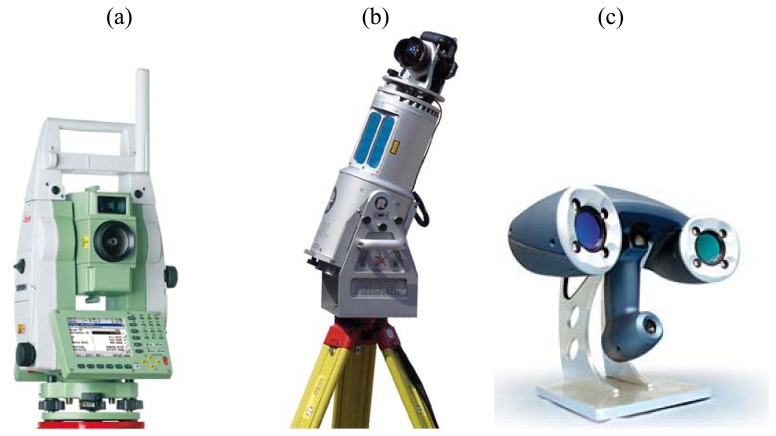
(a) Example of a total station (Leica TPS1200+ Total Station); (b**)** example of a 3D time of flight laser scanner (Riegl LMS-Z420i); (c) example of a 3D triangulation laser scanner (Creaform Handyscan 3D).

Surveying techniques help determine accurately the terrestrial or 3D space position of points and the distances and angles between them, using a total station instrument (Figure 2a) or a laser scanner (Figure 2b).

A laser scanner is usually used to create a point cloud of geometric samples on the surface of an object. These points can then be used to extrapolate the shape of the subject (a process called reconstruction). If color information is collected at each point, then the colors on the surface of the subject can also be determined. In most situations, a single scan will not produce a complete model of the subject. Multiple scans from different directions are therefore required to obtain information about each side of the subject. These scans have to be brought in a common reference system, a process that is usually called alignment or registration, and then merged to create a complete model [7]. Nowadays, laser scanner devices are frequently used for forensic investigation [2], since evidence gathered with laser scanning can be more compelling for juries and allow investigators to virtually “revisit” a crime scene.

Close range photogrammetry allows the acquisition of information about physical objects through the process of recording, measuring, and interpreting photographic images. It may be used in any situation that requires the generation of accurate 3D data and is ideally suited to the survey and measurement of buildings and rooms. Usually, a detailed photogrammetric survey is not carried out immediately after the event, since it is a time-consuming procedure and requires the presence of a skilled technician. However, a post-event survey allows the generation of a map of the crime scene to be used as reference data.

### 2.1. Sole footprint modeling

In order to have a 3D model of a specific sole footprint, required to correctly model human walk as clearly described in Section 4, a high-resolution scan should be acquired. Assuming the availability of the shoes worn by a suspect at the crime scene, a handheld high resolution triangulation scanner can be used in order to generate the model. The instrument used for the acquisition of the shoe sole is the Handyscan 3D produced by Creaform (Figure 2c); this scanner can acquire up to 25,000 points/second with a resolution of about 0.05 mm and an accuracy of 0.04 mm. High resolution 3D survey systems have long existed, especially for mechanical applications. Even if the different available solutions (laser distance measurements on controlled mechanical tables, stand-alone instruments, etc.) are based on different measuring principles, they show common technical problems, related to the stability of the acquisition reference systems during the survey or to the need to have direct access to the object.

The instrument used belongs to a new generation of high resolution and precision scanners which have recently been introduced. With a direct survey in a 3D reference system materialized on the object, or nearby, these new instruments (the so-called third generation scanners) allow a complete surface of convex and concave objects to be scanned avoiding the necessity of moving the object from its natural location and without heavy instrumentation [8]. The basic idea of this instruments is very simple (it is a digital photogrammetric system), but the adopted solutions represent a true novelty from different points of view in the crime scene reconstruction field. The first interesting aspect is the dimension and weight of the acquisition unit which can be handled for long periods without tiring the operator and it allows the possibility of obtaining all the details, even in the case of very complex objects. The second attractive aspect is the possibility of acquiring objects of different dimensions: from small evidences to very large objects [14].

The reference system is materialized onto the shoe soles themselves, by positioning some targets on them. Targets are placed irregularly at a maximum distance of about 15 cm on the soles. A first scan is performed to define the object bounding box and to calibrate the instrument according to the materials the soles are made of. A high accuracy acquisition is then performed. The instrument is able to compute its position in real time, by recognizing the targets it acquires while surveying the object: in this way acquired data are in real time aligned in a unique reference system, the one materialized on the object.

**Figure 3.**
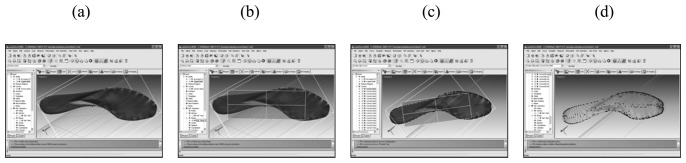
(a) 3D sole model. Stl format; (b) Reference plane; (c) Extraction of Contour lines; (d) Projected Contour Lines.

As an output of this phase, a 3D model of each sole is obtained. The obtained surface (stl format, Figure 3a) is computed by the acquisition system starting from 1 mm spaced points and having a sub-millimetrical precision.

These first models are then cleaned from acquisition noise and errors by using the scanner acquisition software. 3D modeling software can then be used to extract contour lines of the soles. As a reference plane, the one passing through the three main leaning points of the shoe during the walk is considered (Figure 3b). Each leaning point is determined by selecting at least three points on the surface, in order to have a least square computation of the equation of the plane. Contour lines (Figure 3c) are then simplified: just the ones describing areas touching the ground during a walk are kept. A conservative criteria is always adopted in this step, by preferring to be sure to consider areas that completely touch the ground. Simplified Contour lines are then projected to the reference plane (Figure 3d) and used to generate vector polygons in format suitable for the developed GIS procedure.

## 3. Crime scene mapping

In order to correctly define the methodology to be used for the mapping of a crime scene using non metric images, the following considerations have to be taken into account:

1. The goal is to analyze possible walk paths on the crime scene according to the bloodstain pattern on the floor;
2. The object to be represented is the floor covered by the bloodstains. The floor can be considered essentially flat, if the results of the surveying network adjustment show height variations limited to few millimeters;
3. The pictures acquired immediately after a criminal event represent the only available input data. Generally those pictures are acquired for record keeping purposes, thus the attention is focused on the semantic content neglecting the geometric one. Often the viewing direction is not plumbed to the floor, therefore the images are affected by large geometric distortions induced by the perspective;
4. The images are generally acquired with non-metric variable focus cameras and equipped with zoom lenses. Therefore, both the internal orientation parameters of the camera (focal length and position of the principal point) and the lens distortions have to be considered unknown. In the case of non metric cameras the latter can have a value of hundreds of μm in the image plane. Because of the variable focus, these parameters are different for each image acquired with the same camera;
5. Several linear entities are usually recognizable in the images (borders of floor tiles, walls edges, doors/windows edges).

### 3.1. Georeferencing and mosaicking of non metric images

The lack of a calibration certificate (point 3 of the previous list) excludes the possibility of applying a close range photogrammetric approach [19]. In fact, the orthophoto generation, in this case, requires the execution of some self calibration adjustments to define the internal camera calibration parameters (focal length, lens distortions, etc.) and the external orientation parameters for each acquired image (point 4 of the previous list). These self calibrations [15] can be solved using several markers for each images (about 10) through a bundle block adjustment. Fraser *et al.* describing the aforesaid approach applied to the field of accident reconstruction [20], stated that pictures must be taken by a geomatics expert in order to achieve the following goals:

- to generate a complete photo coverage using a single camera with particular lens and a unique focusing;
- to correctly position on the accident scene a sufficient number of markers, whose accurate coordinates have to be surveyed by means of geomatics techniques.

Generally these conditions are not satisfied, because the most important pictures acquired immediately after a criminal event are taken by first aiders and not by a geomatics expert.

Nevertheless, the flatness of the floor (point 2 of the previous list) allows the use of a well known simplified photogrammetric technique, the so called photographic rectification [16, 18].

#### 3.1.1. The lens distortion

The photographic rectification generally neglects the lens distortions since the processed images are acquired with the image plane as parallel (normal case) as possible to the object to be represented. There are numerous commercial software tools that are able to perform a digital rectification in a quasi-normal case, without modeling the lens distortions (ArcGIS by ESRI, ENVI by ITT, Real View, Topko by Sierra Software, etc.). Other rectification software tools (Photometric by GeoPro, MSR by Rollei, etc.) only use camera calibration parameters defined in a specific case (camera, lens and focusing).

**Figure 4.**
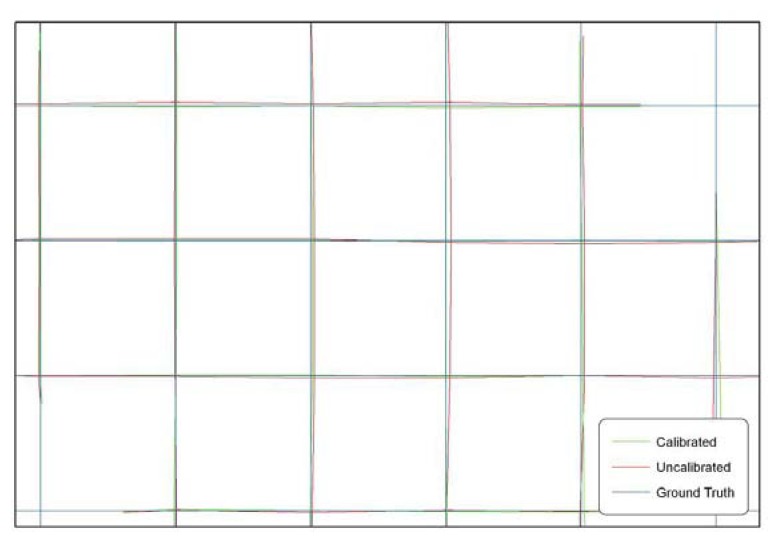
Comparison of rectified images obtained modeling or not modeling the lens radial distortion.

As clearly depicted in the third point of the previous list, the conditions of quasi-normal case and the stability of lens and focusing of the used camera, are generally disregarded during the acquisition of non metric images at a crime scene. Therefore, the application of well-known calibration procedures [15, 17, 19] is not feasible: the lens distortion of each image has to be modeled, especially considering the accuracy requirements of a crime scene reconstruction, using an “*ad hoc*” procedure [21] based on the only available data, that are the non-metric images. Figure 4 shows a test field representing an equally spaced grid of straight lines (blue lines). A picture of the test field has been rectified both neglecting (red lines) and modeling (green lines) the lens radial distortion. Results clearly show the importance of modeling the lens radial distortion to improve the accuracy of the rectified image.

The radial components of the lens distortion have to be estimated for each processed image. This kind of lens distortion causes a given image point to be displaced radially from its desired location on a mean image plane.

According to the goals of the work, the lens radial distortion can be considered a function of the radial distance (ρ) of the object point from the optical axis in the object plane, defined by the following 5^th^ order polynomial:

(1) $$\Delta \rho =k_{1}\rho^{3}+k_{2}\rho^{5}$$

where *k*_1_ and *k*_2_ are the parameters to be determined to model the lens distortion.

In order to estimate these two coefficients, a well known property of the central perspective (used to model the images acquired by frame cameras) is exploited [22]. The perspective projection of straight lines in the object space produces straight lines in the image space in absence of distortions. Photographic images, particularly those acquired with non metric cameras, are characterized by deviations from straightness (sometimes visible to the naked eye) in the image space: this effect can be mainly attributed to lens distortions.

Exploiting this distinctive feature, an “*ad-hoc*” procedure has been developed to estimate *k*_1_ and *k*_2_ radial distortion coefficients, according to the following steps:

- identification of a certain number of straight lines (in the object space) covering the whole image to be calibrated and collimation of the frame coordinates (*ξ*′, *η*′) correspondent to the image coordinates distorted by the lens;
- definition of the straight lines in the ideal image space (not distorted) by the following equation:
  (2) $$\eta =m_{i}\xi +n_{i}$$
  where *i* is the number of the identified straight lines
- definition of the straight lines equation respect to the measured coordinates (*ξ*′, *η*′) by the following:
  (3) $$\eta^{\prime }=m_{i}(\xi^{\prime }+\Delta \xi )+n_{i}-\Delta \eta$$
  where:
  (4) $$\xi =\xi^{\prime }+\Delta \xi$$
  (5) $$\eta =\eta^{\prime }+\Delta \eta$$
  $$\Delta \xi ,\Delta \eta =\Delta \rho \;\text{coordinates},\text{function of}\;k_{1},k_{2};$$
- for each collimated point the equation (3) is linearised respect to the unknowns (*m_i_*, *n_i_*, *k*_1_, *k*_2_) using an approximate solution.
- a least square adjustment is performed, obtaining the desired *k*_1_ and *k*_2_ coefficients
- the analysis of some statistical parameters allows to estimate the adjustment results and the reliability of the estimated coefficients.

#### 3.1.2. Photographic rectification and mosaicking

**Figure 5.**
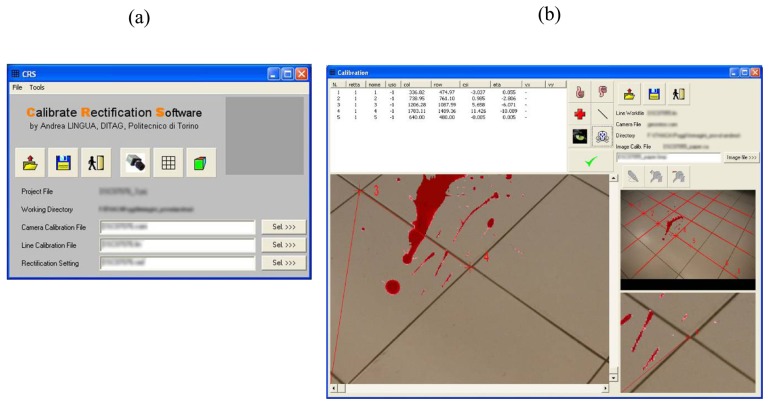
(a) The main window of the developed software CRS; (b) The CRS calibration module: straight lines identification.

Commercial software tools able to carry out the procedure described in 3.1.1 are not currently available in the market. An *ad-hoc* software package, the Calibrate Rectification Software (CRS, Figure 5a), was developed in order to implement the described procedure, using Compaq Visual Fortran libraries and Gino 6.5 graphic routines.

The software is made of two main modules, respectively devoted to the Calibration and Rectification steps.

The Calibration module allows to manually select the straight lines on the processed image (Figure 5b) and subsequently to estimate the lens radial distortion parameters, as clearly described in the previous section.

**Figure 6.**
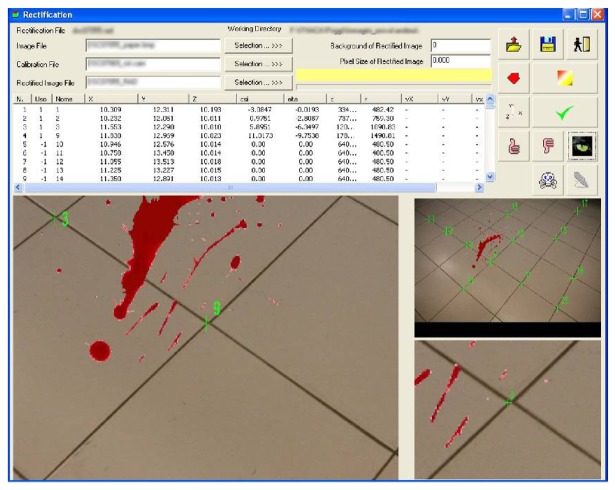
The CRS rectification module: GCP identification.

The Rectification software is aimed at performing the photographic rectification, based on the estimated calibration coefficients. A sufficient number of GCP (coming from the reference data survey described in Section 2) are identified on the image (Figure 6) and their uncorrected image coordinates (*ξ*′, *η*′) are conveniently corrected applying the relations (4) and (5), in function of the estimated lens radial distortion parameters.

The resampling algorithm requires the reverse transformation: since a regular image matrix on the output side (the rectified image) may exist, and for each center of the calculated output pixel, the location in the processed image from which to determine the colour value must be found. Therefore the uncorrected image coordinates are calculated using the following equations:

(6) $$\xi^{\prime }=\xi -\Delta \xi$$

(7) $$\eta^{\prime }=\eta -\Delta \eta$$

**Figure 7.**
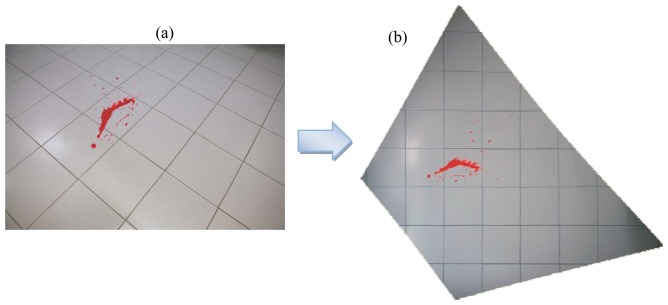
(a) Non metric image; (b) Rectified image.

The rectified images are geometrically corrected and georeferenced in the GCP reference frame, as shown in Figure 7.

Since the crime scene is generally covered by several overlapping images, it is typical that the rectified images are mosaicked to create a seamless image layer. The mosaicked product will consist in a radiometrically balanced, geometrically corrected and digitally blended imagery [3]. Radiometric processing is used to help correct, balance and blend the seams between different input images, since it is quite common that images taken from different cameras under different illumination conditions will require substantial corrections to achieve a consistent mosaic. This product is very useful since it permits extensive and contiguous coverage over large areas, and can be used to measure 2-dimensional coordinates of any object on the scene.

### 3.2. Data processing

As described in the introduction, the main aim of the developed methodology is to analyze the possible movements of suspects in the crime scene, and the eventual relationship with the bloodstains on the floor. The availability of a rectified images mosaic of the crime scene floor (Section 3.1.2), allows to perform a qualitative analysis of the bloodstain pattern. Anyway, the accuracy specifications of a crime scene reconstruction require a quantitative analysis of the bloodstains, aimed at determine the position, the shape and the size of each bloodstain covering the floor.

**Figure 8.**
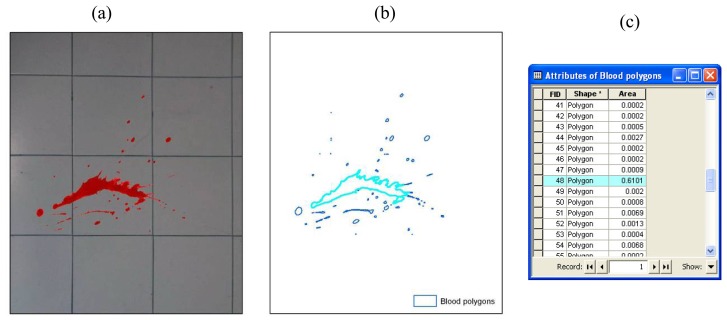
(a) Bloodstain identified on the rectified image; (b) Vector classification of the bloodstain; (c) Attribute table of the blood polygons.

Such goal can be achieved through the classification and vectorization of the blood present in the rectified images, as shown in Figure 8. Supervised classification algorithms, based on the identification of the spectral signature of specific regions of interest, allow to rapidly process the whole image. However the limited number of bands (the photographic pictures of a crime scene are generally taken using amateur cameras that are only sensitive to the visible spectral bands) can effectively limit the classification accuracy, mainly depending on the contrast between the background (the floor) and the feature to be classified (the blood). The variety of shapes and sizes of bloodstains also limits the applicability of objected-oriented classification algorithms.

It is therefore mandatory to perform a thorough photo-interpretation process, aimed at validating and integrating the supervised classification results, which presents the big disadvantage to be a time-consuming procedure. It has to be noticed that the availability of UV images or pictures acquired after the application of a Luminol solution over the area of interest [1], could positively influence the level of automation of the classification process.

## 4. Crime scene analysis

Given a framed space, such as apartment rooms, with a floor covered in bloodstains, the objective of the analysis is to evaluate the probability to walk through the different rooms without any intersection between shoe sole and bloodstain coverage.

**Figure 9.**
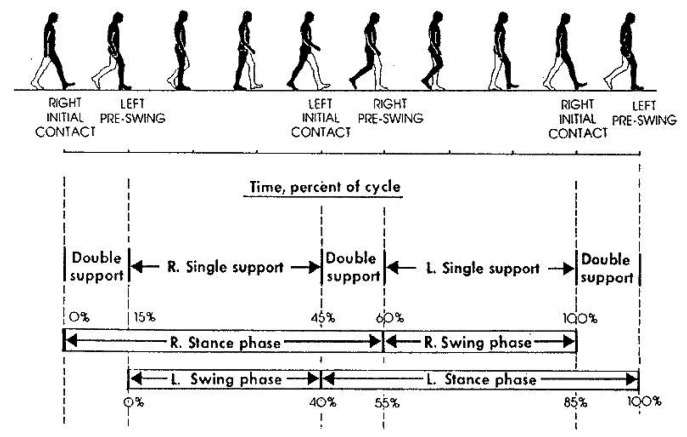
The gait cycle.

The understanding of the biomechanical/anthropometric characteristics responsible for human walk variability is fundamental for the following described approach. The gait cycle is sometimes called the walking cycle. The gait cycle extends from heel strike to heel strike of one leg and includes the stance and swing phases of both legs. In the basic gait cycle the movements are divided into the times when the foot is on the ground (the stance phase which takes up 62% of the full gait cycle) and when the foot is off the ground (the swing phase which takes up the remaining 38% of one full gait cycle) [12] (Figure 9).

**Figure 10.**
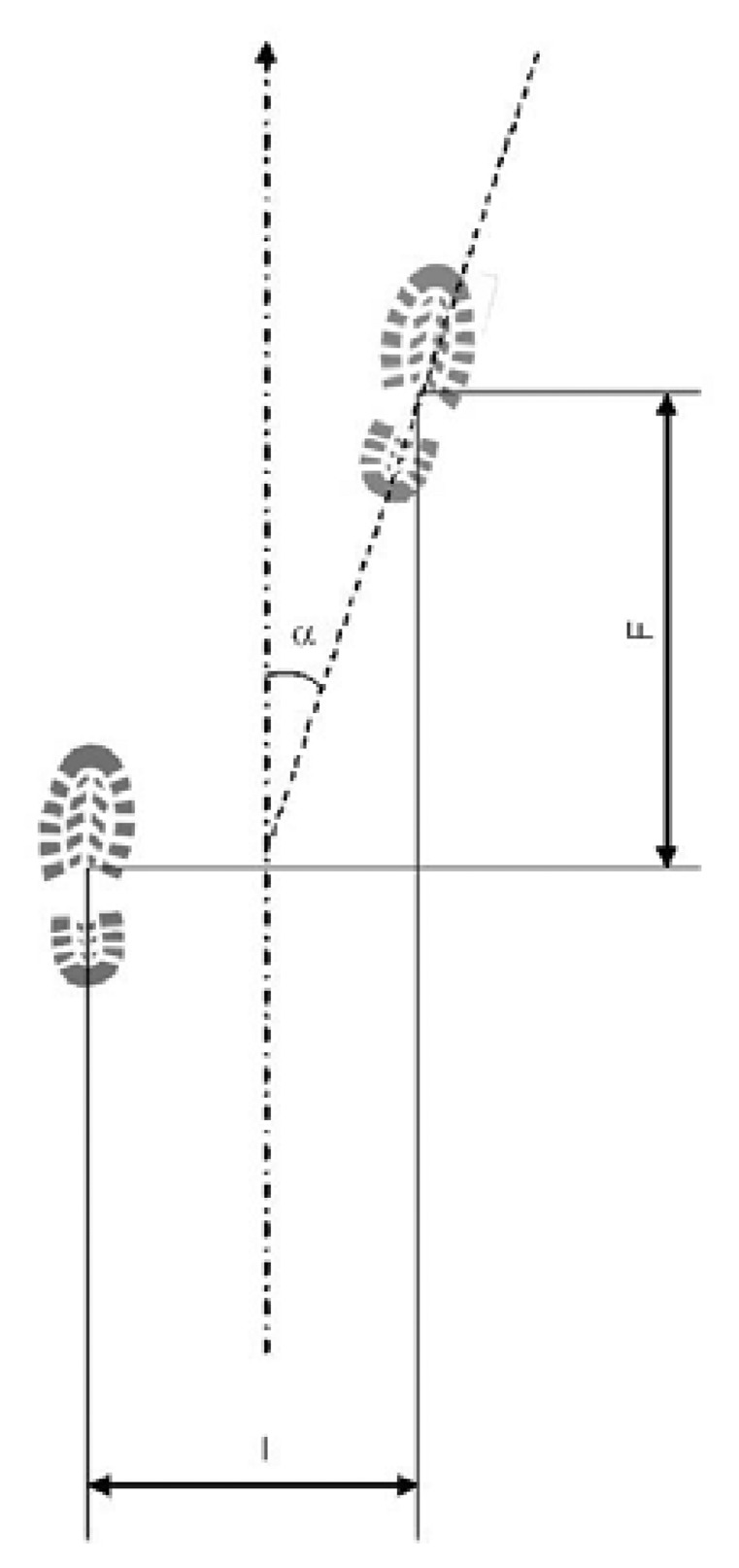
Parameters controlling a human walk.

Based upon literature materials [9, 10, 11, 13], the parameters required for a human walk modeling are (Figure 10):

- Feet distance (I): distance between subsequent steps centroids (barycenters), calculated perpendicularly to walk direction;
- Stride (F): distance between subsequent steps centroids (barycenters), calculated parallel to walk direction;
- Rotation angle (α): step rotation in respect of walk direction.

According to numerous studies based on statistical analysis on significant samples, the three above mentioned parameters in normal conditions may vary between those limits:

- Feet distance (I): 5 – 20 cm;
- Stride (F): 20 – 40 cm (approximately 0.185 × body height in cm);
- Rotation angle (α): 0 – 15°.

The above mentioned three parameters limit the variability of the geometric relation between two subsequent steps, in order to prevent from the generation of unrealistic or impossible step sequences. Those parameters may be kept general or adapted to a specific individual, if available.

### 4.1. Walk simulation and GIS based analysis

**Figure 11.**
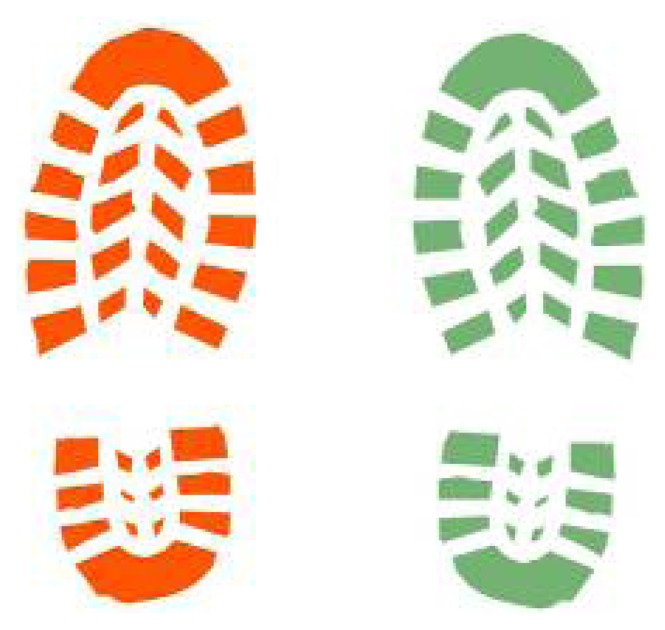
Shoe sole footprints.

Pre-requisites of the analysis are the availability of the shoes in order to reconstruct sole pattern and relative contact surface (Figure 11) on a hard floor, such as a tiled or parquet one. The availability of the real shoes is then critical because particular sole consumption shapes may affect significantly the shape and the extent of the contact surface. Shoe sole are treated as a multi-part polygon in the GIS environment, as the objective is to know if a step (composed by n shoe sole polygons) touches or shares a common geometric part with one or more bloodstains.

The objective of the analysis is to create a large number of randomly generated foot walks in order to evaluate statistically the possibility to walk inside a closed environment, such as apartment rooms, without any contact between shoe sole and floor blood patterns. Any single step, and their association in composing a complete walk, must be created and positioned randomly to avoid biased or unrepresentative sample, that may affect the subsequent statistical analysis. In the generated random output sample, any individual step has the same probability to be selected from the theoretically infinite population of possible steps.

Sample numerosity is a key and delicate factor while planning a survey. Being the population of possible walks theoretically infinite, even within the limits of space and biomechanical/anthropometric factors, it is important to correctly estimate the dimension that a sample must have in order to be considered representative. Sample numerosity is obtained estimating the variance of the parameters to be evaluated (i.e. the total walk surface divided by the surface obtained by the intersection between walks and bloodstains coverage), the confidence level and the absolute precision requested, according to equation (8) [23]:

(8) $$n=\frac{t^{2}\cdot P_{\exp}\left(1-P_{\exp}\right)}{D^{2}}$$

where:

*n* = sample numerosity;
*t* = Student's *t*-distribution;
*P*_exp_ = expected parameter variance;
*D* = absolute precision required.

Suppose that the objective is to statistically determine the possibility of having a complete walk without intersection with bloodstains. In a conservative way, prevalence of the case of walks intersecting bloodstains can be set to 0.5, generating in that way a big sample. If looking to an estimation having confidence level of 99% and an absolute precision of 0.005 (0.5%), the sample numerosity should be equal to:

$$n=\frac{2.576^{2}\cdot 0.5(1-0.5)}{0.005^{2}}=66358$$

Incrementing the confidence level to 99.9% and the absolute precision to 0.001 (0.1%), the sample numerosity should become:

$$n=\frac{3.291^{2}\cdot 0.5(1-0.5)}{0.001^{2}}=2707670$$

Those numbers clearly indicate the necessity of developing automatic procedures to simulate walks, in accordance with a pre-defined environment and biomechanical/anthropometric parameters, and to extract and summarize walk parameters in a structured form.

**Figure 12.**
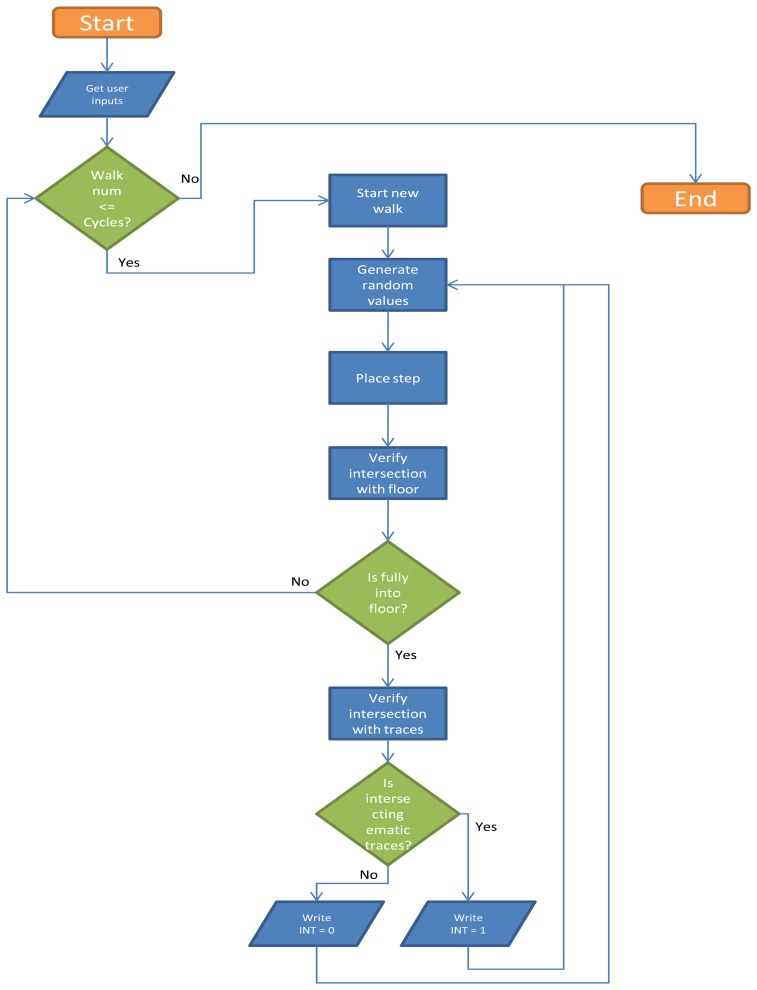
Schematic procedure workflow diagram.

The tool for walk simulation is a completely new and self-developed software component that automatizes the positioning of steps in a pre-defined environment, creating sequences (walks) where single components (steps) are geometrically related each other in a plausible way (according with biomechanical footpath parameters and their variability, described in the introduction of the present chapter) but, at the same time, are generated randomly, in order to produce a statistically significant sample. The analysis of this sample will allow the rigorous definition of the probability of performing a certain walk path without touching any emetic trace (i.e. bloodstains) on floor. The procedures are basically a collection of GIS functionalities for moving objects inside a predefined and measured environment and for verifying any geometrical intersection among them on a 2D environment (Figure 12).

Basic custom inputs for the analysis process are the following parameters:

1. The on floor delimitation of blood patterns (see Section 3.1.3), with possibility of storing total intersection surface for any single step;
2. Left and right footprints derived from the 3D model of the shoe (see Section 2), considering uniquely the part of the shoe sole that touch a flat floor surface while walking. Sole hollows and non-flat surface are not considered while performing geometric intersections among objects;
3. The start line of the foot walk, including all the assumed possible extent where first footprint may have occurred;
4. The floor area, assumed as the possible walking area (optional). In the specific case it is the area resulting from the removal out of the floor surface of all not walkable areas;
5. Minimum and maximum values for distances between feet;
6. Minimum and maximum values for stride;
7. Minimum and maximum values for rotation angles of every footstep;
8. Number of computing cycles (sample dimension);
9. Name and path for the output file.

**Figure 13.**
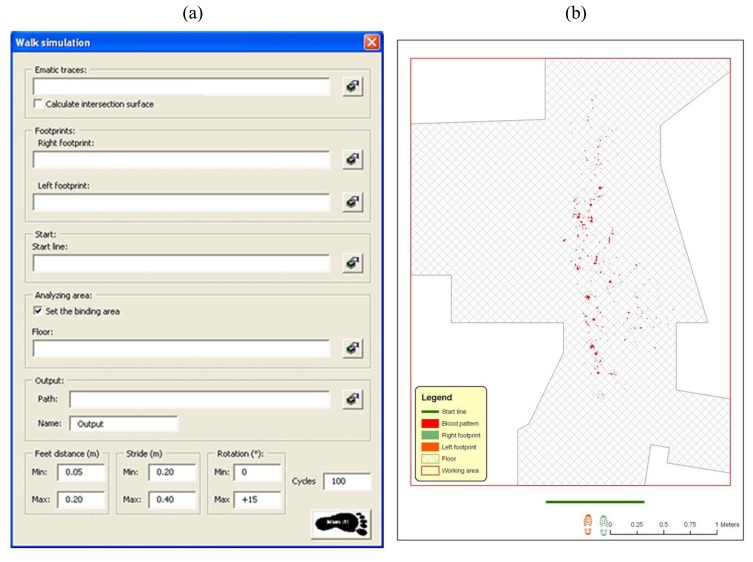
(a) Software input user form. (b) Graphic visualization of sample input datasets.

Software input user form and a graphic representation of input dataset are visualized in Figure 13.

**Figure 14.**
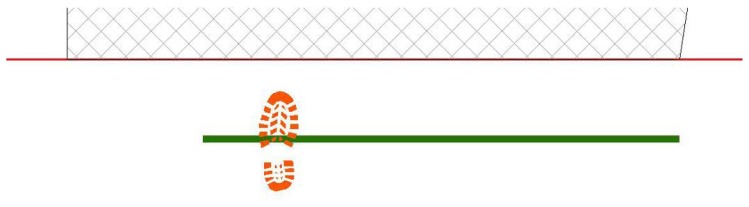
Software workflow, step 1.

**Figure 15.**
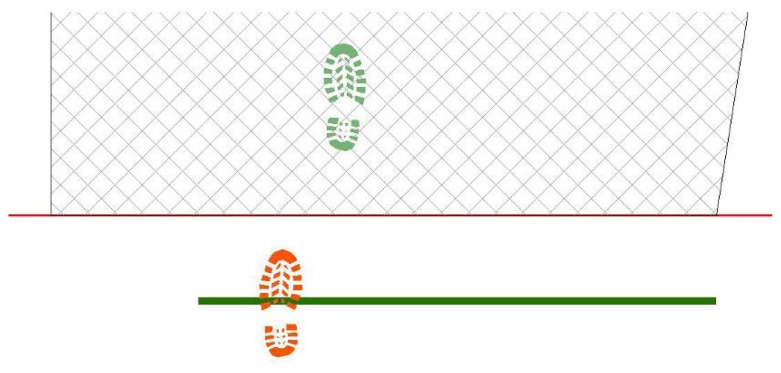
Software workflow, step 2.

**Figure 16.**
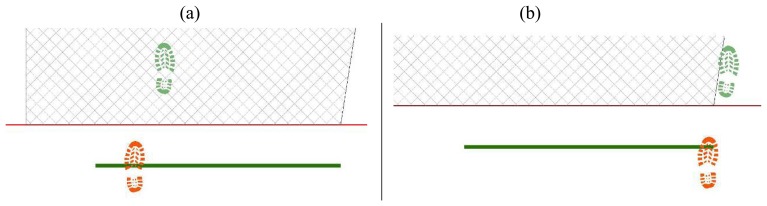
Software workflow, step 3; (a) Step fully contained in floor area; (b) Step totally or partially outside floor area.

**Figure 17.**
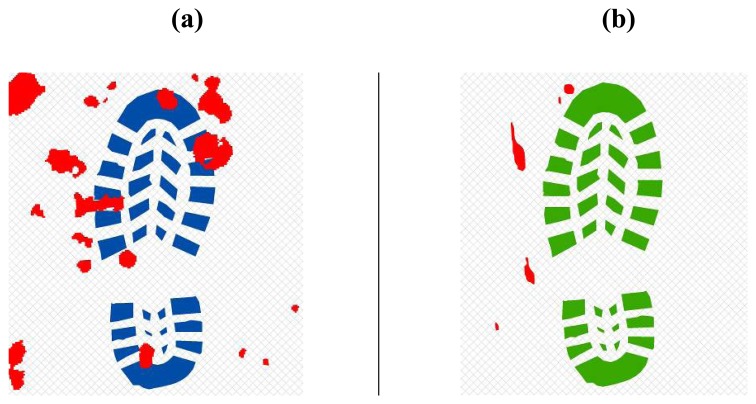
Software workflow, step 4; (a) Step intersecting blood pattern; (b) step non intersecting blood pattern.

**Figure 18.**
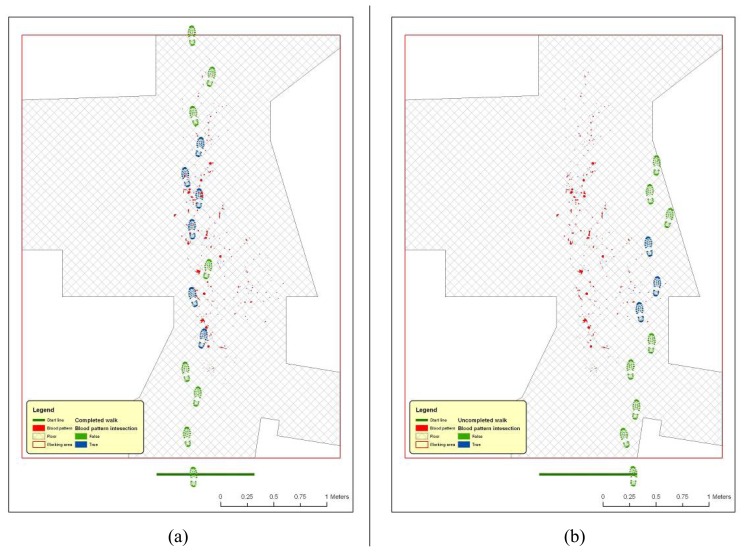
Software workflow, step 5. (a) Completed walk, with computed intersection with bloodstains.;(b) uncompleted walks, with computed intersection with bloodstains.

Application workflow, whose scheme is shown in Figure 12, can be described by the following steps:

1. First step is placed, casually choosing right or left footprint and in proximity of start line, in a casually chosen location (Figure 14);
2. Next step values for feet distance, stride and angles are casually generated (according with limit values defined by the user) and new step position is calculated. According with that positioning information, a new feature is generated, using alternate, left or right, footprint shape (Figure 15). All positional parameters (center x and center y coordinates, feet distance, stride and angle) are stored in the associated table;
3. (Optional) Intersection with floor area is performed (Figure 16). If footprint is fully contained by floor area **(a)**, then procedure continues running. Otherwise (b), a new walk is generated (point 1). Intersection process result is stored in the associated table;
4. Intersection with bloodstains is performed; intersection is verified when any part of a single multi-polygon geometry describing a step shares a common part with any polygon describing the ematic traces. Intersection process result is stored in the associated table. If intersection is verified (Figure 17a), the number of emetic traces intersected and the superimposition area (optional) are calculated and stored in the associated table. Otherwise (Figure 17b) the number of emetic traces intersected and the superimposition area are set to 0;
5. Steps 2 to 4 are repeated until a new step overpass the top limit of the floor area (Figure 18). If walk loop concludes (no 3(b) events during walk generation process), all steps composing the walk are marked. If one or more steps of the walk has intersected bloodstains, the entire walk is marked. That help in easily discriminate entire walks in function of their main characteristics;
6. Steps 1 to 5 are repeated until the number of walks is equal to the number of cycles decided by the user.

### 4.2. Outputs and results analysis

**Figure 19.**
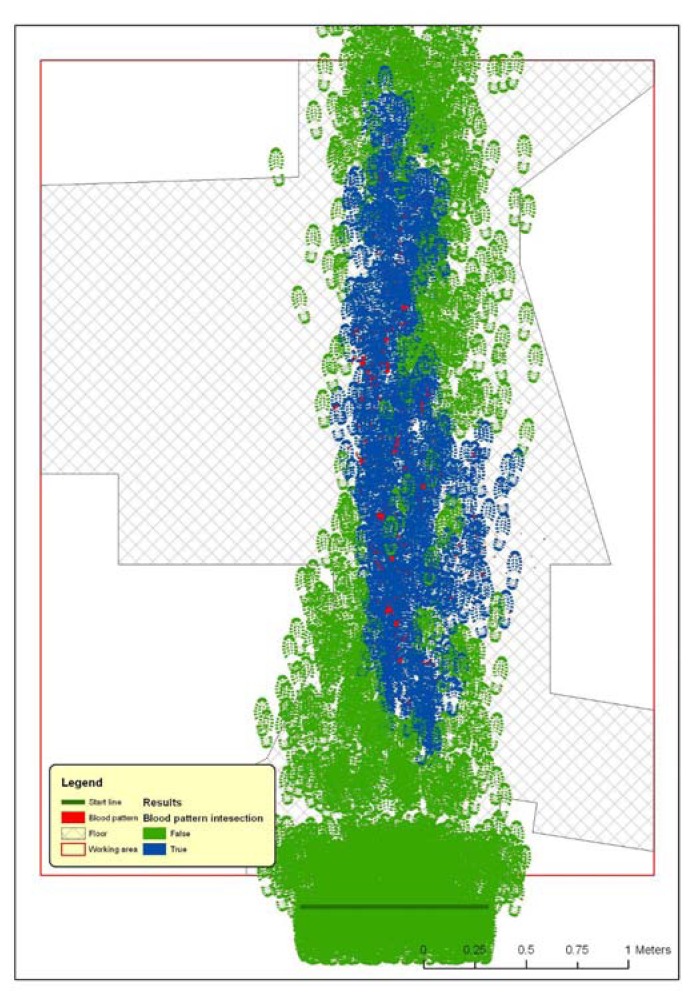
Representation of the result of walk simulation procedure.

At single step level, a feature dataset is produced, containing all steps placed during the execution of the procedure (Figure 19). The associated table contains the attributes listed and described in Table 1.

A table summarized by walks is also produced, containing the attributes listed and described in Table 2. This second table is derived from the one described in Table 1 applying the following SQL statement:
*SELECT Walk, count(Walk) as Cnt_Walk, sum(IntArea) as Sum_IntArea,* *sum(W_IntFloor) as Sum_W_IntFloor, sum(IntAreaVal) as Sum_IntAreaVal,* *sum(Shape_Area) as Sum_Shape_Area, avg(IntAreaNum) as Ave_IntAreaNum,* *avg(IntAreaVal) as Ave_IntAreaVal**FROM [Table1]**GROUP BY Walk;*

This fully automated procedure allows the production of huge amounts of simulated data. Any single walk step is casually placed, in conformity with limit values derived from literature and that any user can adapt for specific situations.

Any intersection between shoe sole and blood patterns is rigorously determined, with the possibility of reporting the 2D dimension of the resulting intersecting area; that parameter may be useful in case of the assumption that small bloodstains and small contact surfaces may not leave detectable residuals on shoe sole

The statistical analysis of such structured outputs would allow to produce reports indicating, in a quantitative way, the possibility to perform a certain path inside a closed area without any geometric intersections between shoe sole and blood patterns.

## 5. Conclusions

The accurate recording of crime scene details is crucial, but it is not always easy to reconcile the need for investigators to document the scene as it is found, with as little as possible external contamination, with the objective of a rigorous reconstruction of the crime scene for virtual revisits or quantitative analysis.

The geomatic approach proposed in that article allows the use of documental material produced in the very first moment after crime discover, acquired with different models of non metric cameras and affected by large geometric distortions induced by the perspective, in order to produce models and to rigorously derive specific metric information, such as blood patterns shapes and position.

Based on those rigorous reconstructions, GIS based analysis procedures may be realized in order to simulate movements on the crime scene and interactions between body parts and the environment. A specific case is the one of a room floor covered by bloodstains and the possibility of a person to walk on the room without touching any blood covered portion of the floor.

Sole characteristics and accurate biomechanical/anthropometric parameters are a key for the accuracy of the results. The development of fully automated procedures allows in a very short time to generate casual and numerous statistical samples for analysis. It will allow generation of several movement scenarios, with different walk paths, room occupation, shoe sole shapes and human behaviors.

The metric approach allows not only to perform true/false analysis, but also to obtain measured parameters, crucial for dimensioning any interaction event. For example, in case of detectable blood traces on shoe sole, bloodstain dimension and presumed elapsed time between bloodstain formation and sole contact must be considered as additional input parameter.

## Figures and Tables

**Table 1.** Single steps attributes and their description.

**Field Name**	**Alias Name**	**Type**	**Length**	**Not Null**
**FID**	FID	OID	4	Yes
**Shape**	Shape	Geometry	0	No
**Id**	Step ID	Integer	4	No
**Walk**	Walk ID	Integer	4	No
**Step**	Step sequence number in Walk	Integer	4	No
**Feet**	Right or Left feet	String	5	No
**XCenter**	X coordinate of the feature center	Double	8	No
**YCenter**	Y coordinate of the feature center	Double	8	No
**Rot**	Rotation angle	Double	8	No
**Stride**	Stride	Double	8	No
**Distance**	Feet distance	Double	8	No
**IntArea**	Intersection with bloodstains (1=true, 2=false)	Small Integer	2	No
**IntFloor**	Intersection with floor (1=true, 2=false)	Double	8	No
**IntAreaVal**	Feet surface touching bloodstains	Double	8	No
**IntAreaNum**	Number of intersected bloodstains	Small Integer	2	No
**W IntArea**	Walk intersection with bloodstains (1=true, 2=false)	Small Integer	2	No
**W IntFloor**	Walk Intersection with floor (1=true, 2=false)	Small Integer	2	No
**Shape Length**	Shape Length	Double	8	Yes
**Shape_Area**	Shape Area	Double	8	Yes

**Table 2.** Walk attributes and their description.

**Field Name**	**Alias Name**	**Type**	**Length**	**Not Null**
**OBJECTID**	FID	OID	4	Yes
**Walk**	Walk ID	Integer	4	Yes
**Cnt_Walk**	N. of steps composing Walk	Integer	4	Yes
**Sum_IntArea**	Total number of steps intersecting bloodstains	Integer	4	Yes
**Sum_W_IntFloor**	N. of steps completely inside the floor area	Integer	4	Yes
**Sum_IntAreaVal**	Total surface covered by bloodstains	Double	8	Yes
**Sum_Shape_Area**	Total walk surface	Double	8	Yes
**Ave_IntAreaNum**	Average number of steps intersecting bloodstains, in comparison with total n. of steps	Double	8	Yes
**Ave_IntAreaVal**	Average area intersecting bloodstains, in comparison with total area	Double	8	Yes
